# Aqua­(2,2′-bipyridine-κ^2^ *N*,*N*′)bis­(4-iodo­benzoato-κ*O*)copper(II)

**DOI:** 10.1107/S1600536810044995

**Published:** 2010-11-10

**Authors:** Mohamad Isa Mohamadin, Norbani Abdullah, Kong Mun Lo, Seik Weng Ng

**Affiliations:** a Department of Chemistry, University of Malaya, 50603 Kuala Lumpur, Malaysia

## Abstract

The Cu^II^ atom in the title compound, [Cu(C_7_H_4_IO_2_)_2_(C_10_H_8_N_2_)(H_2_O)], is *N*,*N*′-chelated by a 2,2′-bipyridine ligand and is coordinated by two monodentate carboxyl­ate ions and a water mol­ecule in a distorted square-pyramidal geometry. The apical site is occupied by one of the carboxyl­ate O atoms. The water mol­ecule forms intra­molecular hydrogen bonds to the uncoordinated carboxyl O atoms. The crystal studied was a nonmerohedral twin with minor components in 0.381 (3) and 0.108 (2) proportions.

## Related literature

For related copper carboxyl­ate–2,2′-bipyridine adducts, see: He *et al.* (2007 ▶); Li *et al.* (2006 ▶); Liu *et al.* (2006 ▶); Yang *et al.* (1994 ▶).
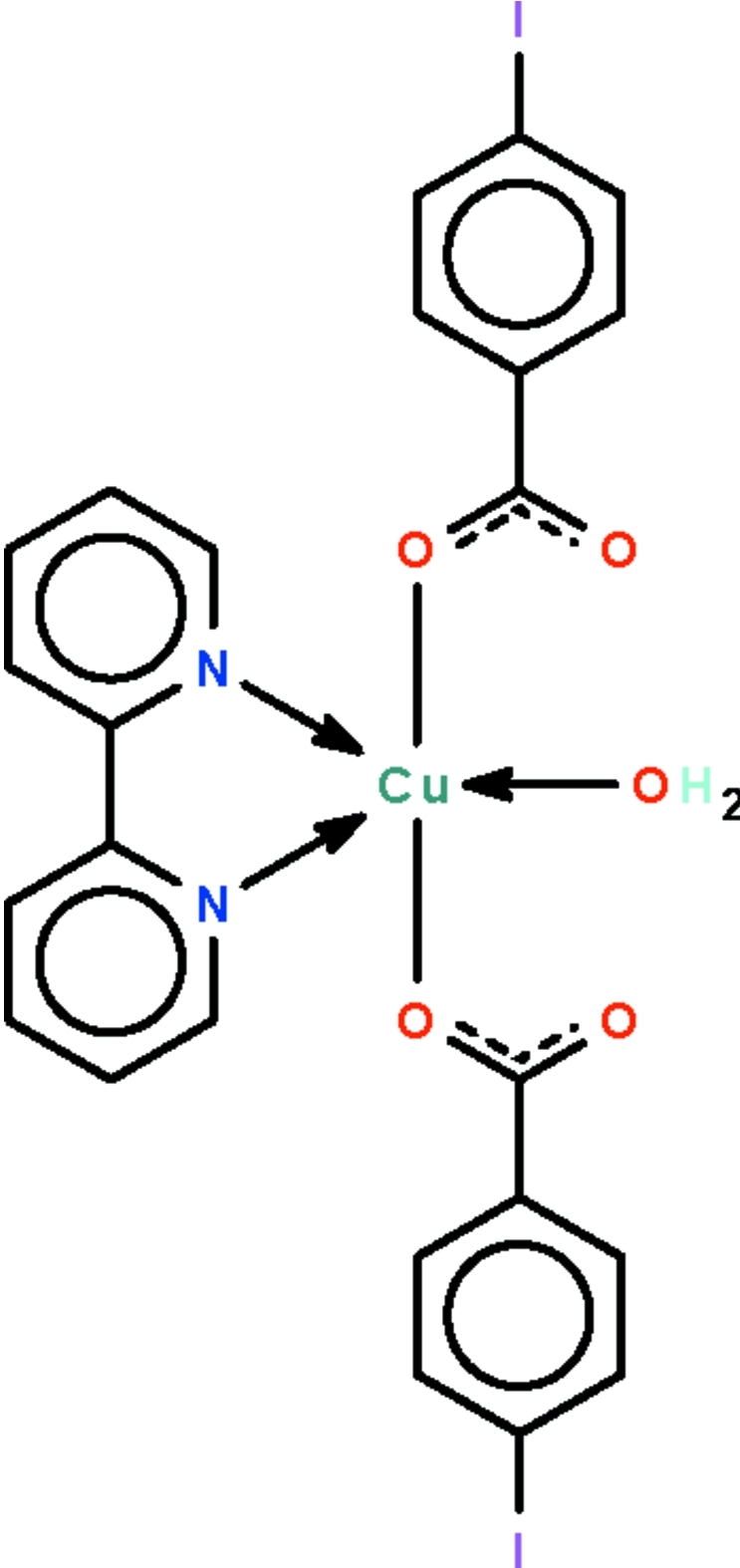


## Experimental

### 

#### Crystal data

[Cu(C_7_H_4_IO_2_)_2_(C_10_H_8_N_2_)(H_2_O)]
*M* *_r_* = 731.74
Monoclinic, 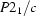

*a* = 13.0571 (2) Å
*b* = 16.0724 (2) Å
*c* = 11.9605 (2) Å
β = 103.298 (1)°
*V* = 2442.72 (6) Å^3^
*Z* = 4
Mo *K*α radiation
μ = 3.46 mm^−1^
*T* = 100 K
0.30 × 0.30 × 0.30 mm

#### Data collection

Bruker SMART APEX diffractometer
Absorption correction: multi-scan (*TWINABS*; Bruker, 2009 ▶) *T*_min_ = 0.355, *T*_max_ = 0.745
33957 measured reflections
5698 independent reflections
4505 reflections with *I* > 2σ(*I*)
*R*_int_ = 0.092

#### Refinement

*R*[*F* ^2^ > 2σ(*F* ^2^)] = 0.058
*wR*(*F* ^2^) = 0.175
*S* = 1.08
5698 reflections
310 parameters
H-atom parameters constrained
Δρ_max_ = 1.71 e Å^−3^
Δρ_min_ = −1.69 e Å^−3^

### 

Data collection: *APEX2* (Bruker, 2009 ▶); cell refinement: *SAINT* (Bruker, 2009 ▶); data reduction: *SAINT*; program(s) used to solve structure: *SHELXS97* (Sheldrick, 2008 ▶); program(s) used to refine structure: *SHELXL97* (Sheldrick, 2008 ▶); molecular graphics: *X-SEED* (Barbour, 2001 ▶); software used to prepare material for publication: *publCIF* (Westrip, 2010 ▶).

## Supplementary Material

Crystal structure: contains datablocks global, I. DOI: 10.1107/S1600536810044995/xu5068sup1.cif

Structure factors: contains datablocks I. DOI: 10.1107/S1600536810044995/xu5068Isup2.hkl

Additional supplementary materials:  crystallographic information; 3D view; checkCIF report

## Figures and Tables

**Table 1:** Selected bond lengths (Å)

Cu1—N1	1.982 (7)
Cu1—N2	2.009 (6)
Cu1—O1	1.977 (5)
Cu1—O3	2.216 (6)
Cu1—O1*W*	1.959 (5)

**Table 2:** Hydrogen-bond geometry (Å, °)

*D*—H⋯*A*	*D*—H	H⋯*A*	*D*⋯*A*	*D*—H⋯*A*
O1w—H11⋯O2	0.84	1.79	2.560 (9)	152
O1w—H12⋯O4	0.84	1.79	2.575 (8)	154

**Fig. 1.**
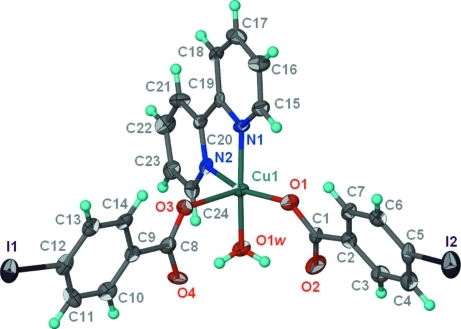
Thermal ellipsoid plot (Barbour, 2001) of Cu(H2O)(C10H8N2)(C7H4IO2)2 at the 70% probability level; hydrogen atoms are drawn as spheres of arbitrary radius.

## supplementary crystallographic information

### Comment

Copper(II) benzoate and its analogs form adducts with 2,2'-bipyridine. The copper benzoate homolog furnishes a water-coordinated adduct (Yang *et al.*, 1994), as does copper *p*-toluate (Li *et al.*, 2006; Liu *et al.*, 2006) and copper *o*-toluate (He *et al.*, 2007). The copper atom in Cu(H_2_O)(C_10_H_8_N_2_)(C_7_H_4_IO_2_)_2_ (Scheme I) is chelated by the *N*-heterocycle and is coordinated by two monodentate carboxylate ions and a water molecule in a square-pyramidal geometry (Fig. 1). The apical site is occupied by the O atom of the carboxylate unit. The crystal studied is a non-merohedral twin with minor components in a 0.381 (3) and 0.108 (2) proportion.

### Experimental

Copper acetate monohydrate (2.00 g, 10 mmol) and 4-iodobenzoic acid (4.96 g, 20 mmol) were heated in aqueous ethanol (1:1, 60 ml) for 1 h. The solvent was removed to give blue copper bis(4-iodobenzoate), which was isolated in 50% yield. The powder and 2,2'-bipyridine (0.77 g, 5 mmol) were dissolved in tetrahydrofuran. Crystals were isolated after several days.

### Refinement

H atoms were placed in calculated positions (C–H 0.95, O–H 0.84 Å) and were included in the refinement in the riding model approximation, with *U*(H) set to 1.2 to 1.5*U*(C,O).

The final difference Fourier map had a peak and a hole in the vicinity of I2.

The crystal studied is a non-merohedral twin with minor components in a 38.1 (3) and 10.8 (2)% proportion. The twinned nature of the crystal structure adversely affected the quality of the diffraction measured, and this is reflected in the somewhat larger numbers in the weighting scheme.

### Crystal data

**Table:**

[Cu(C_7_H_4_IO_2_)_2_(C_10_H_8_N_2_)(H_2_O)]	*F*(000) = 1404
*M**_r_* = 731.74	*D*_x_ = 1.990 Mg m^−^^3^
Monoclinic, *P*2_1_/*c*	Mo *K*α radiation, λ = 0.71073 Å
Hall symbol: -P 2ybc	Cell parameters from 1215 reflections
*a* = 13.0571 (2) Å	θ = 2.5–24.2°
*b* = 16.0724 (2) Å	µ = 3.46 mm^−^^1^
*c* = 11.9605 (2) Å	*T* = 100 K
β = 103.298 (1)°	Cube, blue
*V* = 2442.72 (6) Å^3^	0.30 × 0.30 × 0.30 mm
*Z* = 4	

### Data collection

**Table:**

Bruker SMART APEX diffractometer	5698 independent reflections
Radiation source: fine-focus sealed tube	4505 reflections with *I* > 2σ(*I*)
graphite	*R*_int_ = 0.092
ω scans	θ_max_ = 25.1°, θ_min_ = 2.0°
Absorption correction: multi-scan (*TWINABS*; Bruker, 2009)	*h* = −15→15
*T*_min_ = 0.423, *T*_max_ = 0.423	*k* = 0→19
33957 measured reflections	*l* = 0→14

### Refinement

**Table:**

Refinement on *F*^2^	Primary atom site location: structure-invariant direct methods
Least-squares matrix: full	Secondary atom site location: difference Fourier map
*R*[*F*^2^ > 2σ(*F*^2^)] = 0.058	Hydrogen site location: inferred from neighbouring sites
*wR*(*F*^2^) = 0.175	H-atom parameters constrained
*S* = 1.08	*w* = 1/[σ^2^(*F*_o_^2^) + (0.087*P*)^2^ + 12.0788*P*] where *P* = (*F*_o_^2^ + 2*F*_c_^2^)/3
5698 reflections	(Δ/σ)_max_ = 0.001
310 parameters	Δρ_max_ = 1.71 e Å^−^^3^
0 restraints	Δρ_min_ = −1.69 e Å^−^^3^

### Fractional atomic coordinates and isotropic or equivalent isotropic displacement parameters (Å^2^)

**Table:**

	*x*	*y*	*z*	*U*_iso_*/*U*_eq_	
I1	1.52100 (5)	0.77933 (4)	0.96178 (5)	0.0307 (2)	
I2	0.54254 (5)	1.06313 (4)	0.81444 (6)	0.0370 (2)	
Cu1	0.88404 (8)	0.70624 (6)	0.44393 (8)	0.0165 (3)	
O1	0.7975 (5)	0.7791 (3)	0.5186 (5)	0.0189 (13)	
O2	0.7765 (5)	0.8878 (4)	0.3970 (6)	0.0319 (16)	
O3	1.0368 (4)	0.7208 (3)	0.5695 (5)	0.0182 (12)	
O4	1.0982 (5)	0.8248 (4)	0.4776 (5)	0.0220 (13)	
O1W	0.9172 (5)	0.7943 (3)	0.3446 (5)	0.0203 (13)	
H11	0.8773	0.8352	0.3455	0.030*	
H12	0.9801	0.8092	0.3687	0.030*	
N1	0.8380 (5)	0.6058 (4)	0.5151 (6)	0.0162 (15)	
N2	0.9245 (5)	0.6191 (4)	0.3416 (5)	0.0144 (14)	
C1	0.7637 (7)	0.8513 (5)	0.4846 (8)	0.0215 (19)	
C2	0.7064 (7)	0.8978 (5)	0.5618 (7)	0.0209 (18)	
C3	0.6627 (7)	0.9757 (5)	0.5274 (8)	0.024 (2)	
H3	0.6656	0.9967	0.4540	0.029*	
C4	0.6158 (7)	1.0224 (6)	0.5975 (8)	0.028 (2)	
H4	0.5884	1.0759	0.5737	0.034*	
C5	0.6090 (7)	0.9913 (5)	0.7019 (8)	0.0236 (19)	
C6	0.6510 (7)	0.9127 (5)	0.7390 (8)	0.026 (2)	
H6	0.6470	0.8919	0.8122	0.031*	
C7	0.6977 (7)	0.8665 (5)	0.6682 (7)	0.0219 (19)	
H7	0.7245	0.8128	0.6916	0.026*	
C8	1.1036 (7)	0.7747 (5)	0.5605 (7)	0.0195 (18)	
C9	1.2009 (7)	0.7822 (5)	0.6583 (7)	0.0171 (17)	
C10	1.2850 (7)	0.8289 (5)	0.6483 (7)	0.0217 (19)	
H10	1.2808	0.8612	0.5809	0.026*	
C11	1.3773 (7)	0.8306 (5)	0.7346 (7)	0.0222 (19)	
H11A	1.4365	0.8623	0.7263	0.027*	
C12	1.3794 (7)	0.7842 (5)	0.8332 (7)	0.0216 (19)	
C13	1.2941 (7)	0.7393 (5)	0.8471 (8)	0.0235 (19)	
H13	1.2969	0.7096	0.9164	0.028*	
C14	1.2052 (7)	0.7373 (5)	0.7612 (7)	0.0204 (18)	
H14	1.1460	0.7059	0.7703	0.024*	
C15	0.7940 (7)	0.6048 (5)	0.6056 (7)	0.0218 (19)	
H15	0.7804	0.6566	0.6378	0.026*	
C16	0.7670 (8)	0.5324 (5)	0.6550 (8)	0.025 (2)	
H16	0.7364	0.5339	0.7198	0.030*	
C17	0.7866 (7)	0.4578 (6)	0.6057 (8)	0.026 (2)	
H17	0.7687	0.4068	0.6365	0.031*	
C18	0.8320 (7)	0.4570 (5)	0.5122 (8)	0.0230 (19)	
H18	0.8456	0.4059	0.4782	0.028*	
C19	0.8573 (6)	0.5329 (5)	0.4690 (7)	0.0152 (17)	
C20	0.9038 (6)	0.5399 (5)	0.3673 (7)	0.0166 (17)	
C21	0.9213 (7)	0.4734 (5)	0.3003 (8)	0.026 (2)	
H21	0.9065	0.4181	0.3200	0.031*	
C22	0.9603 (8)	0.4886 (6)	0.2050 (7)	0.026 (2)	
H22	0.9734	0.4439	0.1582	0.031*	
C23	0.9802 (7)	0.5701 (5)	0.1779 (8)	0.024 (2)	
H23	1.0054	0.5821	0.1112	0.029*	
C24	0.9627 (6)	0.6336 (5)	0.2493 (7)	0.0195 (18)	
H24	0.9784	0.6892	0.2320	0.023*	

### Atomic displacement parameters (Å^2^)

**Table:**

	*U*^11^	*U*^22^	*U*^33^	*U*^12^	*U*^13^	*U*^23^
I1	0.0258 (3)	0.0370 (4)	0.0256 (4)	0.0053 (3)	−0.0018 (2)	−0.0007 (2)
I2	0.0303 (4)	0.0424 (4)	0.0393 (4)	0.0066 (3)	0.0099 (3)	−0.0150 (3)
Cu1	0.0216 (6)	0.0126 (5)	0.0153 (5)	−0.0010 (4)	0.0045 (4)	0.0007 (4)
O1	0.026 (3)	0.016 (3)	0.015 (3)	0.000 (2)	0.007 (3)	−0.004 (2)
O2	0.039 (4)	0.029 (4)	0.032 (4)	0.010 (3)	0.017 (3)	0.017 (3)
O3	0.017 (3)	0.020 (3)	0.015 (3)	−0.002 (2)	0.000 (2)	0.000 (2)
O4	0.025 (3)	0.021 (3)	0.019 (3)	−0.007 (3)	0.003 (3)	0.008 (2)
O1W	0.025 (3)	0.017 (3)	0.018 (3)	0.000 (2)	0.002 (3)	0.001 (2)
N1	0.020 (4)	0.010 (3)	0.017 (4)	−0.003 (3)	0.000 (3)	−0.001 (3)
N2	0.016 (3)	0.017 (4)	0.008 (3)	0.003 (3)	0.000 (3)	0.001 (3)
C1	0.016 (4)	0.020 (5)	0.028 (5)	0.004 (3)	0.005 (4)	0.007 (4)
C2	0.017 (4)	0.020 (4)	0.025 (5)	−0.001 (3)	0.002 (4)	0.000 (3)
C3	0.025 (5)	0.023 (5)	0.026 (5)	0.000 (4)	0.008 (4)	0.004 (3)
C4	0.022 (5)	0.026 (5)	0.036 (6)	0.001 (4)	0.006 (4)	−0.003 (4)
C5	0.015 (4)	0.026 (5)	0.027 (5)	0.003 (4)	−0.001 (4)	−0.011 (4)
C6	0.021 (5)	0.022 (5)	0.036 (6)	−0.001 (4)	0.009 (4)	0.001 (4)
C7	0.017 (4)	0.017 (4)	0.027 (5)	0.001 (3)	−0.007 (4)	−0.002 (3)
C8	0.023 (5)	0.015 (4)	0.022 (5)	0.003 (4)	0.008 (4)	−0.004 (3)
C9	0.020 (4)	0.019 (4)	0.014 (4)	0.004 (3)	0.005 (3)	0.002 (3)
C10	0.030 (5)	0.024 (5)	0.013 (4)	0.002 (4)	0.009 (4)	0.000 (3)
C11	0.019 (4)	0.032 (5)	0.017 (4)	−0.005 (4)	0.007 (4)	−0.006 (3)
C12	0.029 (5)	0.022 (4)	0.015 (4)	0.009 (4)	0.007 (4)	−0.008 (3)
C13	0.026 (5)	0.019 (4)	0.025 (5)	0.005 (4)	0.004 (4)	−0.001 (3)
C14	0.025 (5)	0.012 (4)	0.024 (5)	0.004 (3)	0.006 (4)	−0.002 (3)
C15	0.024 (5)	0.017 (4)	0.024 (5)	0.000 (4)	0.004 (4)	−0.001 (3)
C16	0.034 (5)	0.023 (5)	0.019 (5)	−0.004 (4)	0.008 (4)	0.002 (3)
C17	0.028 (5)	0.027 (5)	0.023 (5)	−0.006 (4)	0.008 (4)	0.006 (4)
C18	0.019 (5)	0.013 (4)	0.037 (5)	0.003 (3)	0.006 (4)	0.003 (4)
C19	0.010 (4)	0.019 (4)	0.015 (4)	0.004 (3)	−0.002 (3)	0.004 (3)
C20	0.016 (4)	0.014 (4)	0.019 (5)	0.001 (3)	0.000 (3)	0.000 (3)
C21	0.031 (5)	0.017 (5)	0.029 (5)	−0.005 (4)	0.006 (4)	0.002 (3)
C22	0.035 (5)	0.023 (5)	0.020 (5)	0.002 (4)	0.007 (4)	−0.006 (4)
C23	0.023 (5)	0.028 (5)	0.020 (5)	0.002 (4)	0.002 (4)	0.004 (4)
C24	0.019 (4)	0.025 (5)	0.017 (5)	0.002 (4)	0.009 (4)	−0.001 (3)

### Geometric parameters (Å, °)

**Table:**

I1—C12	2.117 (9)	C8—C9	1.521 (12)
I2—C5	2.106 (8)	C9—C10	1.358 (12)
Cu1—N1	1.982 (7)	C9—C14	1.416 (11)
Cu1—N2	2.009 (6)	C10—C11	1.395 (12)
Cu1—O1	1.977 (5)	C10—H10	0.9500
Cu1—O3	2.216 (6)	C11—C12	1.390 (12)
Cu1—O1W	1.959 (5)	C11—H11A	0.9500
O1—C1	1.275 (10)	C12—C13	1.369 (13)
O2—C1	1.246 (10)	C13—C14	1.363 (12)
O3—C8	1.251 (10)	C13—H13	0.9500
O4—C8	1.268 (10)	C14—H14	0.9500
O1W—H11	0.8400	C15—C16	1.387 (12)
O1W—H12	0.8400	C15—H15	0.9500
N1—C15	1.338 (11)	C16—C17	1.386 (12)
N1—C19	1.344 (10)	C16—H16	0.9500
N2—C24	1.332 (10)	C17—C18	1.380 (12)
N2—C20	1.351 (10)	C17—H17	0.9500
C1—C2	1.513 (12)	C18—C19	1.394 (11)
C2—C7	1.396 (12)	C18—H18	0.9500
C2—C3	1.397 (12)	C19—C20	1.484 (12)
C3—C4	1.369 (12)	C20—C21	1.386 (12)
C3—H3	0.9500	C21—C22	1.373 (12)
C4—C5	1.367 (13)	C21—H21	0.9500
C4—H4	0.9500	C22—C23	1.389 (13)
C5—C6	1.409 (12)	C22—H22	0.9500
C6—C7	1.370 (12)	C23—C24	1.383 (12)
C6—H6	0.9500	C23—H23	0.9500
C7—H7	0.9500	C24—H24	0.9500
			
O1W—Cu1—O1	94.3 (2)	C14—C9—C8	119.1 (7)
O1W—Cu1—N1	168.6 (3)	C9—C10—C11	121.6 (8)
O1—Cu1—N1	91.6 (3)	C9—C10—H10	119.2
O1W—Cu1—N2	90.5 (3)	C11—C10—H10	119.2
O1—Cu1—N2	161.0 (3)	C12—C11—C10	117.5 (8)
N1—Cu1—N2	80.9 (3)	C12—C11—H11A	121.3
O1W—Cu1—O3	92.6 (2)	C10—C11—H11A	121.3
O1—Cu1—O3	98.7 (2)	C13—C12—C11	121.9 (8)
N1—Cu1—O3	96.3 (2)	C13—C12—I1	119.4 (6)
N2—Cu1—O3	99.5 (2)	C11—C12—I1	118.6 (7)
C1—O1—Cu1	126.0 (5)	C14—C13—C12	119.8 (8)
C8—O3—Cu1	123.5 (5)	C14—C13—H13	120.1
Cu1—O1W—H11	109.5	C12—C13—H13	120.1
Cu1—O1W—H12	109.5	C13—C14—C9	120.0 (8)
H11—O1W—H12	109.5	C13—C14—H14	120.0
C15—N1—C19	118.6 (7)	C9—C14—H14	120.0
C15—N1—Cu1	125.9 (5)	N1—C15—C16	123.6 (8)
C19—N1—Cu1	115.5 (5)	N1—C15—H15	118.2
C24—N2—C20	119.1 (7)	C16—C15—H15	118.2
C24—N2—Cu1	125.7 (6)	C17—C16—C15	117.1 (8)
C20—N2—Cu1	115.1 (5)	C17—C16—H16	121.5
O2—C1—O1	126.4 (8)	C15—C16—H16	121.5
O2—C1—C2	117.5 (7)	C18—C17—C16	120.6 (8)
O1—C1—C2	116.1 (7)	C18—C17—H17	119.7
C7—C2—C3	118.5 (8)	C16—C17—H17	119.7
C7—C2—C1	122.2 (8)	C17—C18—C19	118.3 (8)
C3—C2—C1	119.3 (7)	C17—C18—H18	120.9
C4—C3—C2	121.4 (8)	C19—C18—H18	120.9
C4—C3—H3	119.3	N1—C19—C18	121.9 (7)
C2—C3—H3	119.3	N1—C19—C20	114.9 (7)
C5—C4—C3	119.4 (9)	C18—C19—C20	123.2 (7)
C5—C4—H4	120.3	N2—C20—C21	121.8 (7)
C3—C4—H4	120.3	N2—C20—C19	113.5 (7)
C4—C5—C6	120.9 (8)	C21—C20—C19	124.6 (7)
C4—C5—I2	120.5 (7)	C22—C21—C20	119.0 (8)
C6—C5—I2	118.6 (6)	C22—C21—H21	120.5
C7—C6—C5	119.2 (8)	C20—C21—H21	120.5
C7—C6—H6	120.4	C21—C22—C23	119.1 (8)
C5—C6—H6	120.4	C21—C22—H22	120.4
C6—C7—C2	120.6 (8)	C23—C22—H22	120.4
C6—C7—H7	119.7	C24—C23—C22	119.1 (8)
C2—C7—H7	119.7	C24—C23—H23	120.5
O3—C8—O4	126.4 (8)	C22—C23—H23	120.5
O3—C8—C9	117.7 (7)	N2—C24—C23	121.9 (8)
O4—C8—C9	115.9 (7)	N2—C24—H24	119.0
C10—C9—C14	119.1 (8)	C23—C24—H24	119.0
C10—C9—C8	121.7 (7)		
			
O1W—Cu1—O1—C1	13.0 (7)	Cu1—O3—C8—C9	174.9 (5)
N1—Cu1—O1—C1	−157.2 (7)	O3—C8—C9—C10	169.1 (8)
N2—Cu1—O1—C1	−91.2 (10)	O4—C8—C9—C10	−11.3 (11)
O3—Cu1—O1—C1	106.2 (7)	O3—C8—C9—C14	−9.0 (11)
O1W—Cu1—O3—C8	2.8 (6)	O4—C8—C9—C14	170.5 (7)
O1—Cu1—O3—C8	−91.9 (6)	C14—C9—C10—C11	3.1 (12)
N1—Cu1—O3—C8	175.6 (6)	C8—C9—C10—C11	−175.0 (7)
N2—Cu1—O3—C8	93.8 (6)	C9—C10—C11—C12	−1.6 (12)
O1W—Cu1—N1—C15	−138.5 (12)	C10—C11—C12—C13	−1.0 (12)
O1—Cu1—N1—C15	−17.7 (7)	C10—C11—C12—I1	176.3 (6)
N2—Cu1—N1—C15	179.8 (7)	C11—C12—C13—C14	2.0 (12)
O3—Cu1—N1—C15	81.2 (7)	I1—C12—C13—C14	−175.3 (6)
O1W—Cu1—N1—C19	44.0 (17)	C12—C13—C14—C9	−0.5 (12)
O1—Cu1—N1—C19	164.8 (6)	C10—C9—C14—C13	−2.1 (12)
N2—Cu1—N1—C19	2.3 (6)	C8—C9—C14—C13	176.1 (7)
O3—Cu1—N1—C19	−96.3 (6)	C19—N1—C15—C16	0.1 (13)
O1W—Cu1—N2—C24	2.9 (7)	Cu1—N1—C15—C16	−177.3 (7)
O1—Cu1—N2—C24	107.7 (9)	N1—C15—C16—C17	−0.6 (14)
N1—Cu1—N2—C24	175.3 (7)	C15—C16—C17—C18	0.6 (14)
O3—Cu1—N2—C24	−89.8 (7)	C16—C17—C18—C19	0.0 (14)
O1W—Cu1—N2—C20	−172.7 (6)	C15—N1—C19—C18	0.5 (12)
O1—Cu1—N2—C20	−68.0 (10)	Cu1—N1—C19—C18	178.2 (6)
N1—Cu1—N2—C20	−0.3 (6)	C15—N1—C19—C20	178.5 (7)
O3—Cu1—N2—C20	94.6 (6)	Cu1—N1—C19—C20	−3.8 (9)
Cu1—O1—C1—O2	2.1 (13)	C17—C18—C19—N1	−0.6 (13)
Cu1—O1—C1—C2	−175.2 (5)	C17—C18—C19—C20	−178.4 (8)
O2—C1—C2—C7	−172.9 (8)	C24—N2—C20—C21	0.1 (12)
O1—C1—C2—C7	4.7 (12)	Cu1—N2—C20—C21	176.0 (7)
O2—C1—C2—C3	5.4 (13)	C24—N2—C20—C19	−177.5 (7)
O1—C1—C2—C3	−177.0 (8)	Cu1—N2—C20—C19	−1.5 (9)
C7—C2—C3—C4	2.5 (13)	N1—C19—C20—N2	3.5 (11)
C1—C2—C3—C4	−175.9 (9)	C18—C19—C20—N2	−178.6 (8)
C2—C3—C4—C5	−1.8 (14)	N1—C19—C20—C21	−174.0 (8)
C3—C4—C5—C6	1.1 (14)	C18—C19—C20—C21	4.0 (14)
C3—C4—C5—I2	177.7 (7)	N2—C20—C21—C22	−0.3 (14)
C4—C5—C6—C7	−1.1 (13)	C19—C20—C21—C22	176.9 (8)
I2—C5—C6—C7	−177.8 (7)	C20—C21—C22—C23	−0.6 (14)
C5—C6—C7—C2	1.7 (13)	C21—C22—C23—C24	1.6 (14)
C3—C2—C7—C6	−2.4 (13)	C20—N2—C24—C23	1.1 (13)
C1—C2—C7—C6	175.9 (8)	Cu1—N2—C24—C23	−174.4 (6)
Cu1—O3—C8—O4	−4.6 (12)	C22—C23—C24—N2	−1.9 (14)

### Hydrogen-bond geometry (Å, °)

**Table:**

*D*—H···*A*	*D*—H	H···*A*	*D*···*A*	*D*—H···*A*
O1w—H11···O2	0.84	1.79	2.560 (9)	152
O1w—H12···O4	0.84	1.79	2.575 (8)	154
